# Reach and implementation of human and AI-assisted diabetic retinopathy screening models in primary healthcare settings in India

**DOI:** 10.1038/s41598-025-25402-9

**Published:** 2025-11-21

**Authors:** Anshul Chauhan, Luke Vale, Ankita Kankaria, Vishali Gupta, Mandeep Singh, Gagandeep Kaur, Sonam Kumar, Mitesh Yadav, Neha N, Basavaraj Tigari, Sanjay Bhadada, Mona Duggal

**Affiliations:** 1 Advanced Eye Centre (AEC), Post Graduate Institute of Medical Education and Research, Chandigarh, 160012 India; 2 Global Health Economics Centre, London School of Hygiene and Tropical Medicine, Tavistock Place, London, UK; 3 Department of Community Medicine and School of Public Health, Post Graduate Institute of Medical Education and Research, Chandigarh, 160012 India; 4 Department of Endocrinology, Post Graduate Institute of Medical Education and Research, Chandigarh, 160012 India; 5 National Institute for Research in Digital Health and Data Science (NIRDHDS), India Council of Medical Research (ICMR), New Delhi, Delhi 110029 India

**Keywords:** Diabetic retinopathy screening, Artificial intelligence, Reach, Implementation, RE-AIM, Pragmatic trial

## Abstract

Diabetic retinopathy (DR) is a leading cause of preventable vision loss. While DR screening is critical, evidence on the reach and implementation of different screening models in primary healthcare settings is limited. This study evaluated the reach and implementation of DRS models in northern India using the RE-AIM framework. A pragmatic three-arm observational study was conducted between February 2023 and January 2024 in Block Boothgarh, a rural block in District Mohali, Punjab, comprising 30 villages with an estimated 120,000 residents. Household line listing was performed to identify individuals aged 30 years or older with diabetes. Participants (n = 600) were equally allocated to three screening models: facility-based screening at Health and Wellness Centres (HWC) by non-ophthalmologists, community-based AI-assisted screening at home, and standard care. Reach and implementation were assessed through quantitative data, field observations, and qualitative interviews with healthcare providers. Refusal for screening was higher in facility-based screening (40%, 135/340) and lower in community-based screening (13%, 31/240). Older individuals were more likely to decline participation, with a mean age of 62.0 years for males and 60.3 years for females. Reported barriers included existing medical conditions, mobility limitations, perceived good eye health, travel distance, and transportation difficulties. Concerns regarding long-term medication adherence also reduced uptake. Technical issues, including power outages, hardware or software malfunctions, suboptimal image quality, and lack of cooperation, further declined implementation. Adaptations, including the use of backup power generators, on-site troubleshooting, and provision of transport support, mitigated these barriers and improved overall implementation fidelity. Assessing reach is essential for the success of public health interventions. Using the RE-AIM framework, this study identified key barriers and adaptive strategies in DRS, enhancing both reach and implementation within primary healthcare settings. These findings can inform the integration of DRS models into comparable resource-constrained contexts, thereby improving overall effectiveness.

*Clinical Trial Registry of India (CTRI)*: 2022/10/046283.

**Supplementary Information:**

The online version contains supplementary material available at 10.1038/s41598-025-25402-9.

## Introduction

Diabetes mellitus (DM), a prevalent chronic disease, is associated with systemic complications, including diabetic retinopathy (DR)^1^. In India, the estimated national prevalence of DR is 12.5% (95% CI: 11.0–14.2), with vision-threatening DR (VTDR) affecting 4.0% (95% CI: 3.4–4.8). DR prevalence is 15.5% (95% CI: 13.4–17.8) in known DM versus 8.0% (95% CI: 6.3–10.1) in undiagnosed DM; VTDR is 5.3% (95% CI: 4.5–6.3) versus 2.4% (95% CI: 1.6–3.6), respectively^1^. DR is often asymptomatic in its early stages but can progress to VTDR without timely detection and treatment. Identifying all patients with VTDR is therefore critical, given the high burden of DM Untreated VTDR leads to severe vision impairment in 26% of individuals within two years^2,3^, highlighting the need for proactive screening of all people with DM for timely detection and management^4^.

Addressing this challenge, the Government of India has implemented population-based screening for common NCDs in line with the WHO Global NCD Action Plan 2013–2020^5^. Within this program, NCD registers systematically track individuals with chronic conditions such as DM, providing a mechanism to facilitate regular DR screening (DRS)^6^. Systematic screening programs that use existing ophthalmic resources are more cost-effective than opportunistic or no screening^7,8^. Primary healthcare settings, where people with DM are routinely managed, provide an appropriate platform to improve DRS coverage^7,9,10^. The availability of ophthalmology services is insufficient to cater to growing healthcare demands^4^. DRS has been proposed to be implemented in community settings and healthcare facilities^6^. Community-based screening brings testing directly to people with DM, increasing access and community engagement^6^. The operational guidelines for primary eye care at Health and Wellness Centres (HWCs) in India recommend DRS and early referral to eye specialists^11^. A well-functioning referral system is critical to ensure continuity of care for DR management^12^. However, the lack of mechanisms to track referral adherence often leaves physicians and ophthalmologists unaware of patient outcomes^12^.

Effective DRS interventions must address multiple levels, including individual and institutional factors^13^. Key barriers in primary care include high workload, limited time, and established organizational culture^9,10,14^. The healthcare system also faces challenges, including insufficient equipment, inefficient referral systems, and limited national policies^15^. Additionally, low awareness and health literacy, financial and transportation constraints, competing priorities, and the asymptomatic nature of DR also impact the screening attendance^15^. Due to the large number of people with DM requiring repeated screenings, no single DRS model can be universally applied^16^.

This study presents the implementation of DRS at 1) HWC, 2) community-based programs, and 3) standard care. It describes the approaches employed to identify people with DM, facilitate their participation, and implement the screening models effectively. The implementation process is evaluated using the RE-AIM framework, which allows analysis of data collected before, during, and after the intervention^17^. In this paper, we focus on the process evaluation, specifically, reach and implementation. The other components, such as effectiveness and adoption outcomes, will be reported separately.

## Methods

This manuscript has been prepared using a template for intervention description and replication (TIDieR) for qualitative studies^18^ (Supplementary table 1).

### Study design and setting

A pragmatic trial^19^ was conducted in Block Boothgarh, district Mohali, Punjab, India, comparing two DRS models with standard care across three parallel, equal-sized arms. The study enrolled 600 PwDM (200 per arm) between February 2023 and January 2024; the protocol is described elsewhere^20^. The study was performed in accordance with the Declaration of Helsinki. It was approved by the Institutional Ethics Committee of the Postgraduate Institute of Medical Education and Research (PGIMER), Chandigarh (PGI/IEC/2020/000741) and registered with the Clinical Trial Registry of India (CTRI: 2022/10/046283).

Punjab, located in northwestern India, has a population of approximately 28 million and is divided into 23 districts. Mohali district, with around 990,000 residents, 55.2% urban, is divided into three community development (CD) blocks: Majri, Kharar, and Dera Bassi^21,22^. The study was conducted in Boothgarh, a health block within Majri, situated 17 km from PGIMER (tertiary health care centre), Chandigarh, and 14 km from the district hospital. District Mohali has approximately 423 villages and includes 76 functioning HWCs, 19 primary health centres (PHCs), five community health centres (CHCs), and a single district hospital^22^ (Supplementary table 2). Block Boothgarh of CD Block Majri has a population of approximately 120,000 and has 14 out of 19 operational HWCs. (PHC records) Three of the 14 operational HWCs in block Boothgarh were randomly selected. Households within each HWC coverage area were line-listed to identify eligible individuals aged 30 and above. Additional HWCs near the already line-listed centres were included to complete the sample size.

The prevalence of DR in Punjab was 12.8%^1^. The existing process of DM management and DRS referral centres in the district of Mohali has been presented in Supplementary Table 3.

### Intervention description and inclusion criteria

The study included individuals aged 30 years and above with diabetes. Exclusion criteria were age below 30 without diabetes, lack of consent, history of intraocular surgery or retinal treatment, and active eye infections or injuries^20^.

Arm I implemented non-ophthalmologist-led DR screening at HWC Khijrabad, while Arm II employed AI-based DRS in community (home) settings. Arm III followed standard care, which involved counselling without DR screening and referral to the DH for ophthalmology review. Participants in Arms I and II were referred to the DH if they had referable DR or ungradable images^23^ (Supplementary Fig. 1).

### Procedures

This manuscript primarily focuses on the methods used to evaluate the reach and implementation domains of the RE-AIM framework.

### Reach (R)

**Fig. 1:**
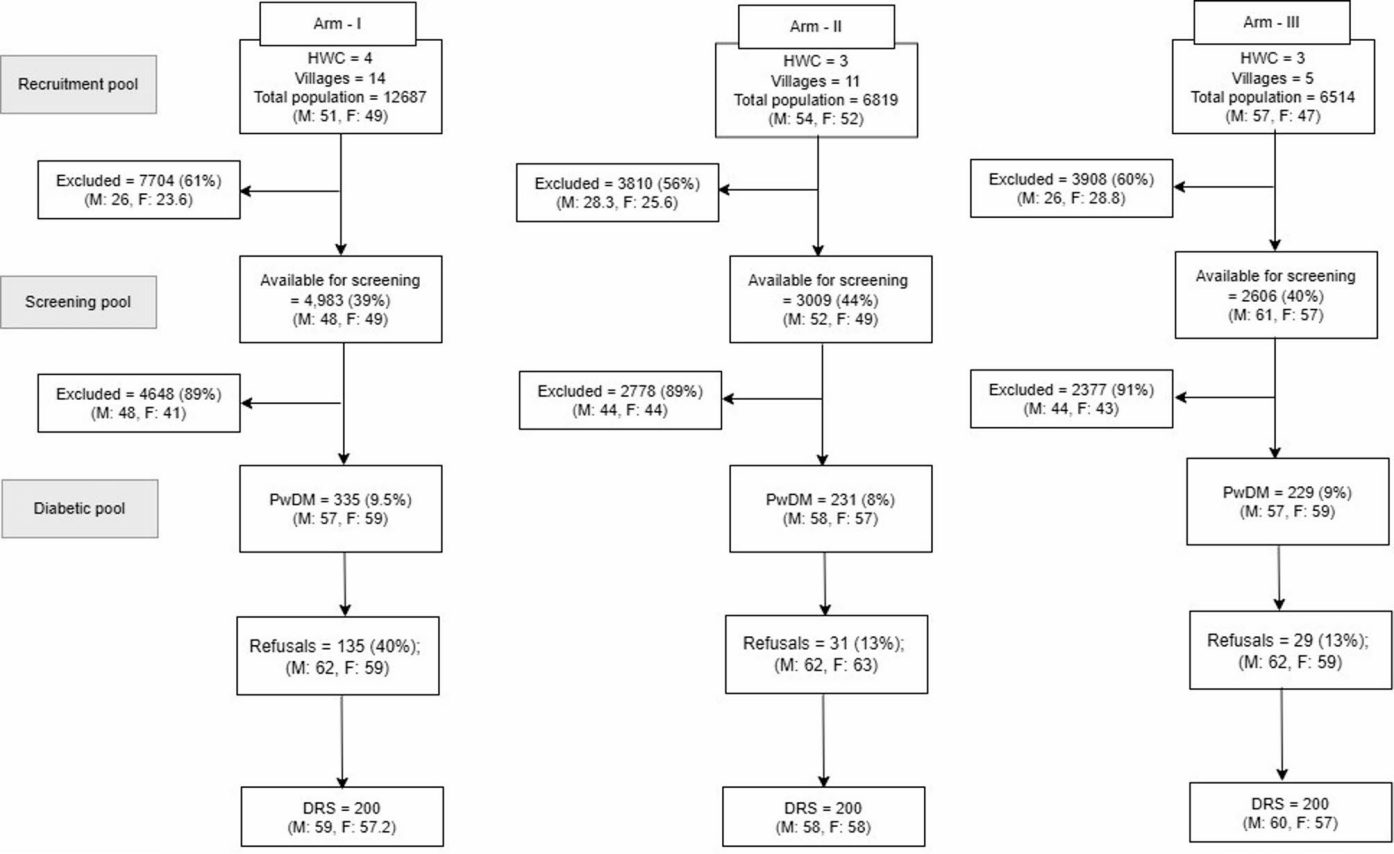
Study flow chart describing reach (R). *DRS: Diabetic retinopathy screening, HWC: Health and wellness centre, M: Male; F: Female. The numbers alongside M and F represent the mean age of the participants.

Reach refers to efforts to identify people with diabetes (PwDM) aged 30 years and above. Field investigators and a supervisor line-listed village households, while a mapper created a village map to aid house identification during follow-up visits^19^. Individuals under 30 were excluded^20^, leaving a screening pool of eligible participants, from which PwDM were identified for the study (Fig. 1).

People with diabetes received an appointment card for DRS in Arms I and II or a counselling session in the standard care arm by the research team during line listing^20^. A reminder call was made by the optometrists a day before, and participants were consecutively enrolled, with reasons for refusal or non-attendance documented.

### Implementation (I)

**Fig. 2:**
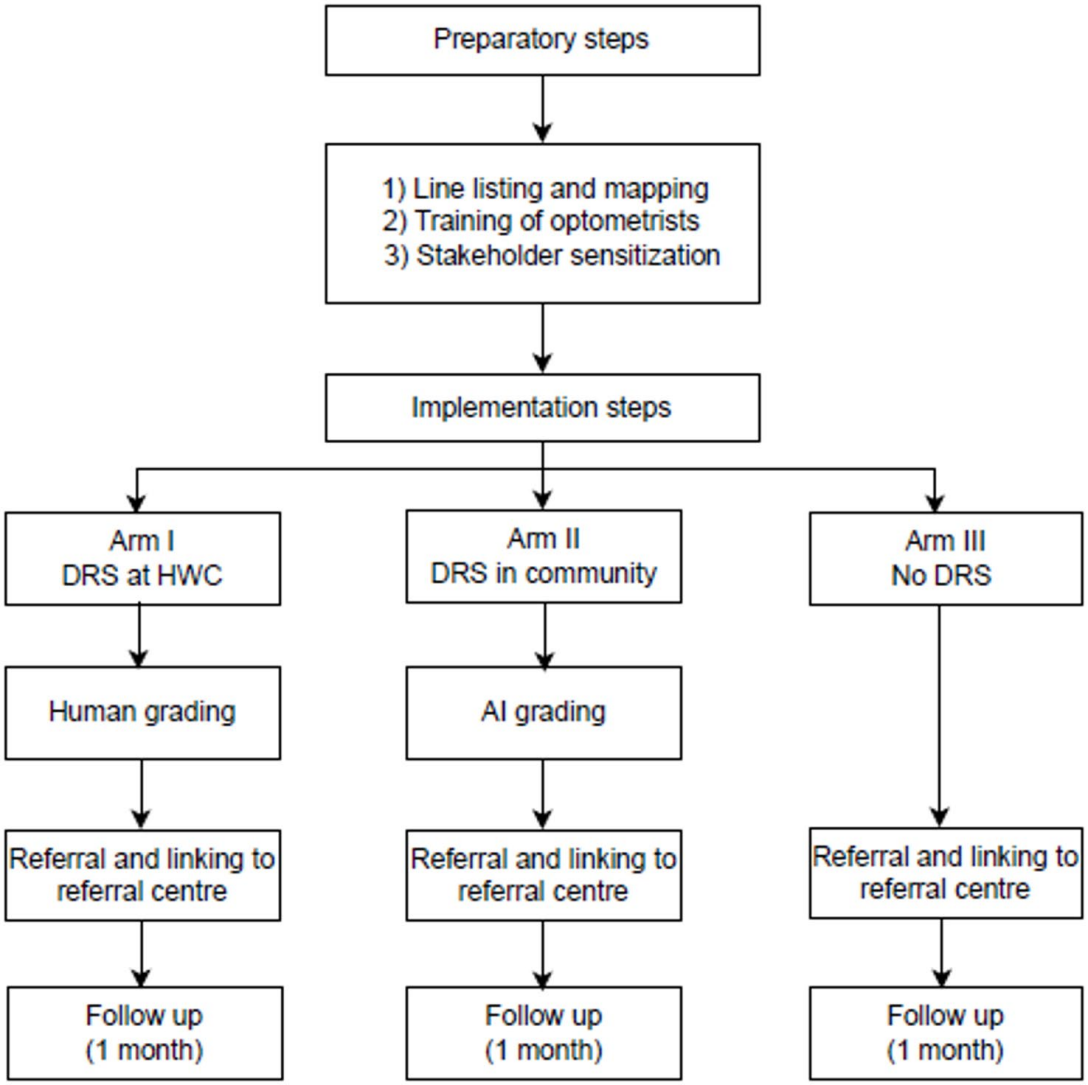
Preparatory and implementation steps of the DRS in study arms. AI: Artificial intelligence, DRS: Diabetic retinopathy screening.

The implementation was evaluated by examining four key dimensions as an outcome, including the intervention delivery and time: (i) intervention delivery, (ii) consistency of implementation, (iii) details of content, and (iv) challenges and adaptations during implementation. The activities flow has been described as preparatory and implementation steps (Fig. 2).

### Preparatory steps

The DRS intervention was evaluated across three arms, with all optometrists trained and accredited to implement the program. Three non-ophthalmologists (hereafter optometrists) captured fundus images following a 15-day training at PGIMER Advanced Eye Centre, which equipped them with the necessary skills. Sensitization meetings at the PHC and village levels introduced the study and secured stakeholder support. Line-listing and mapping procedures are detailed in the protocol paper^20^. See Supplementary Fig. 2 for maps created during the study.

### Diabetic retinopathy screening

Participants sat in the screening room for 4–6 min to achieve physiological mydriasis under low-light conditions. Optometrists captured two fundus images per eye, centered on the macula and disc. Fundus images were captured using the Forus 3nethra classic benchtop camera at HWCs^24,25^. The Remidio NM FOP 10 smartphone-based camera with the offline Medios AI algorithm in community settings^26,27^ (Supplementary Fig. 1). Standard care involved no DRS. Preliminary data, including age, sex, DM duration, and ocular history, were recorded.

### Diabetic retinopathy grading

#### Human grading (reference standard)

In Arm I, fundus images were de-identified and uploaded to Amazon Web Services (AWS) for grading by three independent masked human graders: a certified optometrist (HG1), an ophthalmologist (HG2), and a senior retinal specialist (HG3). HG3 resolved disagreements between HG1 and HG2, which was considered as a reference standard grading to evaluate screening performance.

#### Offline AI grading (Medios)

In Arm II, a smartphone-integrated Medios AI algorithm was used for DR detection^26,28^. The algorithm was operated by the optometrist after image acquisition and provided two outputs: “no signs of DR detected” (non-RDR) and “signs of DR detected” (RDR). All the AI graded images were evaluated by HGs.

#### Report sharing and referral recommendations

Final grading reports were provided to PwDM through ASHA workers. Arm I participants received handwritten reports, while Arm II participants received AI-generated reports within seven days. ASHAs were trained by the research staff (field investigator) to explain the results and referral instructions in Punjabi to participants.

##### Arms I and II

ASHAs counselled participants with no DR or mild NPDR to visit the district hospital within one year. Those with moderate NPDR or ungradable images were referred to the DH for ophthalmology review within one month^6^. The patients with severe NPDR, PDR, or macular edema were advised to visit a tertiary eye institute within one month^29^. These timelines differed from ICO guidelines, which recommend six months for moderate NPDR and three months and one month for severe NPDR and PDR, respectively^30^. This approach allowed the study to assess adherence to a one-month follow-up under limited resources. PwDM with cataract or ungradable images were also referred to as part of the protocol^31^.

##### Arm III

AllPwDM in Arm III were referred to the district hospital for ophthalmology review.

### Counselling after screening

The counselling focused on raising awareness of healthy lifestyles and NCD risk factors, including DM. Optometrists guided PwDM on metabolic control, lifestyle management, medication adherence, follow-up care, and avoidance of tobacco and alcohol, and the importance of early DR detection^32^.

### Linking to referral centres

A referral system linked the referred participants with designated centres, including ophthalmologists at DH Mohali and vitreoretinal consultants at AEC, PGIMER. Participant IDs and DR diagnoses were shared with these contacts, and study staff coordinated follow-up. One month later, participants in all arms were contacted up to three times by telephone to ascertain visit status, with diagnoses recorded for attendees and reasons for non-attendance documented.

### Qualitative interviews to record the Reach and Implementation challenges

#### Reach

In addition, in-depth interviews (IDIs) were conducted with healthcare providers (HCPs) to explore challenges faced by participants in accessing DRS. HCPs included Medical Officers (MOs) from PHCs, Community Health Officers (CHOs) from HWCs, and ASHAs from the study villages (Supplementary Table 4). MOs and CHOs were recruited via convenience sampling and SMO recommendations, while ASHAs were selected on the MO’s recommendation.

#### Implementation

Operational challenges during implementation were systematically documented through field notes, screening flow tracking, and in-depth interviews. Adaptations were iteratively developed, allowing real-time adjustments to address emerging barriers. Data collection focused on real-time observations of workflow barriers and team adaptations. During the preparatory phase, it was observed that uncontrolled lighting in the screening room caused pupil constriction and could compromise image quality. (Supplementary Fig. 3a, 3b,3c, 3d) The screening area was converted into a controlled dark space by blocking daylight with opaque boards, covering windows with black chart paper, painting walls and ceilings matte black, and installing floor-length double-sided black curtains to prevent light infiltration. A Light Meter App (Google Play Store: https://play.google.com/store/apps) was used to ensure zero lux conditions at room corners and the camera’s chin stand.

The principal investigator (PI), project coordinator (PC), ophthalmologist from the district hospital (Oph), optometrist involved in screening (Optom), and ASHA workers’ perspectives were gathered. The PI, PC, and Optom oversaw project implementation, while the ophthalmologist provided clinical support, and the ASHA workers facilitated participant recruitment. Screened participants were also interviewed to explore their challenges in accessing DRS at the HWC. Two experienced research fellows (AC, MS) with expertise in qualitative research conducted face-to-face interviews in quiet locations within the workplace. The PwDM were interviewed in Hindi or Punjabi, while the other group primarily used English. Written informed consent was obtained, and interviews were audio recorded.

### Analysis

#### Quantitative

We analyzed descriptive statistics for participant characteristics, presenting continuous variables as mean (SD) and categorical variables as absolute counts (n) with corresponding percentages. All statistical data were analyzed using Stata SE 15.0 (StataCorp LLC)^33^.

#### Qualitative

The interviews conducted in Hindi and Punjabi were transcribed and translated into English by MS. The research scholar (AC) and trained investigator (MS) familiarized themselves with the data, which were analyzed thematically to identify issues related to DRS access. Key reach-domain themes included 1) proximity to healthcare facilities, 2) transportation availability, and 3) participants’ attitudes toward eye care. Implementation-domain data highlighted challenges and lessons learned during DRS delivery. Two reviewers (AC, AK) coded all texts, with themes discussed among authors (MD), and public health experts, MD and AK merged overlapping codes to avoid redundancy.

Field notes were thematically analyzed and categorized under several key areas: barriers to screening accessibility and community outreach, ergonomic barriers, workflow optimization, addressing infrastructure and equipment limitations, and optimizing image quality through darkroom adaptations. The research fellow (AC) familiarised themselves with the data. Triangulation was performed by cross-validating field notes with in-depth interviews, patient feedback, and quantitative screening data to assess real-time adjustments. Finally, a narrative synthesis linked challenges to solutions, providing contextual insights into the effectiveness of screening.

## Results

The sociodemographic characteristics of the study population (Supplementary table 5). Of the 600 participants, 355 (59%) were female. The mean age was 58.2 ± 11.5 years, with a median of 59 years (IQR: 50–65). Most participants (60%) were aged 51–70 years, and nearly half (48%) had diabetes for ≤ 5 years. Arm I had a notably higher proportion of participants aged 41–50 (26.5%) than Arms II and III (16% and 16.9%, respectively).

The reach section will present the activities and challenges of identifying the PwDM. The implementation section will present a comprehensive overview of the implementation activities included in all study arms.

### Reach (R)

Figure 1 illustrates the line-listing of 30 villages covered by ten HWCs, encompassing a total population of 26,023, which served as the recruitment pool for the study’s three arms. Approximately 7,704 individuals (61%) in Arm I, 3810 individuals (56%) in Arm II, and 3,908 individuals (60%) in Arm III were under 30 years of age and thus excluded from the study. The remaining individuals in each arm formed the screening pool. The screening pool consisted of 4,983 individuals with DM (39%) in Arm I, 3,009 (44%) in Arm II, and 2,606 (40%) in Arm III, with non-diabetic individuals excluded from the study. In Arm I, 335 out of 4,983 individuals with DM (9.5%) were eligible for screening, though 135 (40%) declined participation. In Arms II, 231 out of 3,009 individuals with DM (8%) were eligible, with 31 (13%) opting not to participate. In Arm III, 229 out of 2,606 individuals with DM (9%) were eligible for screening, and 29 (13%) declined participation.

The average age of males and females who refused screening in the study arms is presented in Fig. 1. The primary factor leading to refusal for screening in Arm I was the travel distance, 38/140 (27%), to the screening facility (HWC). The other contributing factors were concurrent medical problems, the inability to cooperate during screening, and the lack of accurate information for household tracing at the time of the screening invitation, consistent across all three study arms. The refusals were predominantly associated with problems with distance and transportation to the screening facilities. (Supplementary Table 3).

Interviews with the HCPs revealed that patients prioritize healthcare facilities based on proximity to their residence, the availability of public transportation or access to personally owned vehicles, and their attitudes and perceptions toward eye care. These logistical considerations significantly influence their ability to seek and access healthcare services. Representative quotes illustrating these findings are presented below.

“They change the health facility as per their distance, availability of public transport, or personally owned vehicle” CHO1

HCPs emphasized that transportation challenges often prevent residents of remote villages from accessing essential medicines at distant local dispensaries.

“Certain villages are located remotely. In some areas where transportation is an issue, people find it challenging even to come to the dispensary to take medicines and forget about eye screening at the farthest locations” MO1

Participants also expressed concern that people often only visit health facilities if faced with an emergency. This behaviour likely contributes to the low participation in preventive initiatives like eye screening programs. The reluctance to seek routine or preventive care reflects a broader issue of limited awareness about early detection and treatment, compounded by logistical and accessibility challenges in some areas.

“The people do not visit until it is an emergency, which might be a reason for fallout in your screening initiative” MO2

Some participants expressed mistrust in medical treatments, sharing concerns that doctors often prescribe multiple medications, which they perceive as causing more harm than good. They reported that instead of providing relief, these medicines sometimes exacerbate their symptoms, leading to dissatisfaction with healthcare services and a reluctance to seek further medical assistance.

“Doctors put you on many medications, which affect you worse, and the medicines do not cause any relief but worsen your symptoms” MO2, CHO2, ASHA1

### Implementation

The results in the implementation domain are presented under the following categories: 1) intervention delivery, 2) consistency of implementation, 3) details of content, and 4) challenges and adaptations during implementation. Steps 1–3 are presented with preparatory steps, Diabetic retinopathy screening and grading, and participant follow-up.

### Preparatory steps: line listing (LL), mapping, and enrolment

Arm I (HWC) covered 2,042 households and 12,687 individuals over 39 days. Daily tasks included a 10-min introduction, 4 min for house numbering, and 12 min for data capture. Arm II and Arm III (Community) covered 1,124 and 1,466 households over 22 and 26 days. Form entry and mapping required 11 days in Arm II and 14 days in Arm III. Field investigators and mappers visited PwDM households to schedule two-day screenings in each arm.(Supplementary Table 6).

### Diabetic retinopathy screening and grading

Comprehensive eye examinations (CEE) lasted 5–6 min in Arm I and ~ 10 min in Arms II and III. Non-mydriatic fundus imaging (NMFI) in Arm I required a shorter time due to the controlled environment. In contrast, community-based screenings in Arms II and III took longer per image than Arm I. Arm I screened 12 PwDM daily; Arms II and III screened 8. Total screening time per participant was 28 min in Arm I and 48 min in Arms II and III. Screening 200 participants required 32 days in Arm II and 34 days in Arm III, due to the need for equipment transport and community-based procedures. HG1 and HG2 graded 12 retinal images daily; HG3 resolved disagreements over a period of two days. Image grading for 200 participants spanned 17 days. ASHA workers distributed results within seven days in each arm. (Supplementary Table 6).

### Participant follow-up

The field investigator followed up after one month in Arms I and II via telephone calls within eight days and in Arm III within 17 days for all 200 participants. (Supplementary Table 7).

### Challenges and adaptations during the implementation

The in-depth interviews and field data are integrated into the results section, where qualitative insights are presented alongside field observations. Direct interview quotes are included to complement and contextualize the findings, aligning with the methodology that combines real-time field documentation with participant perspectives to provide a comprehensive understanding of implementation challenges and adaptations. The representative quotes under changes and adaptations are presented in the Supplementary Table 8.

A total of nine key stakeholders were interviewed, including three males and six females. The group included one PI, one PC, one ophthalmologist, two optometrists, and four ASHA workers. Their mean professional experience was seven years, with an average age of 39.4 ± 8.4 years. Six patients who underwent screening were also interviewed.

### Barriers to screening accessibility and community outreach

Access to screening was constrained by limited mobility, lack of public transport, and reliance on family members, especially among older patients. Awareness barriers also emerged, as many did not perceive screening as urgent in the absence of symptoms. In response, community sensitization meetings, transport arrangements (n = 6 participants), and engagement with local leaders and frontline health workers were introduced. In community-based screening (Arms II–III), multiple household visits were required due to poor network connectivity and the need to match participants’ availability. Cultural barriers also emerged, as women in some villages refused entry to male team members (*“woh ghar me ane se mana kar dete hain”, “They refuse entry into their houses”*). The team adapted by using offline data capture, managing batteries with spares and charging breaks, and including a female researcher for households with female participants. These measures improved both image quality and participation. These adaptations reduced non-attendance and improved participation, highlighting the importance of combining logistical support with community outreach. (Supplementary Table 8).

### Ergonomic barriers and workflow optimization

Difficulties in patient seating and positioning frequently compromised the quality of non-mydriatic images. Operators emphasized that inadequate posture not only delayed the process but also reduced image clarity. Ergonomic modifications were introduced by relocating adjustable chairs and a height-adjustable camera table from the Advanced Eye Centre, which created more stable screening conditions. In parallel, a focused training session led by a certified retina technician strengthened the optometrist’s skills in patient handling, transfer, and positioning. These adjustments minimized ergonomic stressors, lowered the risk of operator injury, and improved the overall efficiency of image acquisition (Table 1).

**Table 1:** Summary of operational challenges and adaptive solutions during diabetic retinopathy screening implementation.

Category	Operational challenge	Adaptation	Outcomes
Barriers to screening accessibility and community outreach	Long travel distances, lack of transport, mobility limitations in the elderly, and low DR awareness	Arranged vehicle support to PHC, ensured wheelchair accessibility, and conducted awareness campaigns with ASHA workers	Increased accessibility for elderly and mobility-restricted patients, improved screening attendance
Ergonomic barriers and workflow optimization	Patient discomfort due to improper posture during imaging, and operator fatigue affecting efficiency	Provided hands-on training for optometrists in patient positioning and ergonomics, introduced motorized tables and height-adjustable chairs	Improved patient positioning, better image capture, reduced operator fatigue, enhanced efficiency
Addressing infrastructure and equipment limitations	Power outages, voltage fluctuations, hardware failures leading to unsaved images, and patient discomfort	Installed voltage stabilizer and generator, procured new chargers, and established remote troubleshooting with the camera engineer	Minimized downtime and ensured uninterrupted screening
Optimizing image quality through darkroom adaptations	Bright lighting conditions cause pupil constriction and poor image quality	Customized darkroom setup. Light levels were optimized using a light meter app	95.6% gradable images Significantly improved image clarity and efficiency

*ASHA: Accredited Social Health Activist, PHC: Primary Health Centre.

### Addressing infrastructure and equipment limitations

The implementation faced significant infrastructure and equipment challenges, mainly due to summer power outages and voltage fluctuations in rural health facilities. These disruptions damaged essential screening equipment, including the camera, laptop adapter, and table cables. As a result, screenings were frequently interrupted, leading to patient frustration and longer wait times. Several participants had to return for repeat visits, increasing their travel burden. Unsaved images sometimes force technicians to re-capture images, further delaying the screening process and reducing efficiency. To ensure uninterrupted screening, the team procured new chargers with compatible voltage and amperage, installed a voltage stabilizer, and arranged a generator as a backup during outages (Table 1).

### Optimizing image quality through darkroom adaptations

During the preparatory phase, 20/80 (26%) non-mydriatic fundus images from 20 participants were ungradable. Following the establishment of a dedicated darkroom, image quality improved substantially: 367 of 400 images (91.8%) were gradable, while only 33 (8.3%) remained ungradable (to be presented elsewhere). The darkroom adaptation was therefore instrumental in enhancing image quality and reducing the need for retakes (Table 1).

Whereas bright ambient lighting in most homes hindered non-mydriatic fundus imaging, so shaded areas or darker rooms were used. Limited camera battery life further constrained screening during long sessions.

## Discussion

The global burden of diabetes and vision loss from DR is increasing, especially in LMICs, highlighting the need for periodic population-based studies^34^. This study applied the RE-AIM framework to guide, execute, and document large-scale DRS interventions in primary healthcare. It enabled detailed evaluation of recruitment (reach) and delivery (implementation), marking the first Indian study to examine these aspects and referral for VTDR treatment at the primary care level^2,3^. In our study HWC-based screening showed high refusal rates (38%), mainly due to facility distance, whereas arms II and III had lower refusal (13%), indicating preference for community-based screening. Barriers preventing PwDM from visiting HWCs included lack of transport, preference for convenient facilities *(“apni marzi se jate hai”, “They go on their own will”),* patient attitudes *(“waiting for an emergency”),* and perceptions about DRS *(“medication apprehension”),* as supported by HCP interviews.

Community outreach with transportation support and wheelchair accessibility enhanced attendance, consistent with evidence that locally tailored strategies strengthen healthcare access in resource-constrained settings^35,36^. Despite these adaptations, variability in infrastructure and workforce capacity across regions may influence scalability.

Patient-related factors, including health beliefs, perceived lack of need, misconceptions about DR, limited awareness of regular screening, time constraints, family support, and socio-economic status^15,37^, were not assessed for reach in this study. Access to rural primary care in LMICs faces inadequate infrastructure, staff shortages, and limited screening equipment, contributing to low uptake^38^. Health system factors were not evaluated, as screening occurred as a research project without active stakeholder involvement^39^. HWCs recommend early DRS screening and timely referral for people with diabetes.

However, implementing the DRS in primary healthcare settings also faces technical challenges, such as power quality issues, non-standard voltage, current, or frequency deviations, which can lead to equipment damage, increased waiting times, and a decline in screening rates^40^. Persistent power outages highlight the need for reliable electricity within India’s public health system, a prerequisite for high-quality service delivery^40^. While addressing these challenges, sensitizing the community members and facilitating travel (mobilizing patients) to the screening facility was crucial in reaffirming participation^41^.

In India, nearly 70% of the population resides in rural areas, and the ophthalmologist-to-patient ratio is low. Offline smartphone-based AI for DR diagnosis enables scalable, community-based screening^42,43^. Ergonomic challenges include awkward postures during patient handling, transfer, and repositioning, contributing to operator stress^44^.

Addressing ergonomic challenges during DRS is vital to minimizing operator fatigue and maintaining high-quality imaging^44^. The Occupational Safety and Health Administration (OSHA) advises reducing ergonomic stressors by utilizing assistive devices and engineering controls whenever feasible^45^. At the HWC, we equipped the screening area with motorized tables and height-adjustable chairs to ensure optimal seating for patients and camera operators, thereby reducing fatigue, improving efficiency, and decreasing image capture time. In contrast, the research optometrist reported difficulties capturing clear images at patients homes due to frequent movements, *“the image doesn’t come out clear if the patient moves” "patient ke hilne pe photo theek na91.8hi aati"*. This extends image capture time to 10–12 min per patient, unlike the shorter times achieved with smartphone-based cameras^46^. Community-based screening encountered challenges such as low device batteries, repeated visits, and longer screening days. The FOP NM camera also struggled with small pupils and media opacities^46^. Screening outcomes, including diagnostic accuracy, referral rates, adherence, and costs, are presented separately.

### Strength

A key strength of this study lies in its first-of-its-kind comparison of DRS models in primary healthcare using a pragmatic trial design and the RE-AIM framework. It captures challenges in recruiting people with diabetes and implementing screening models, emphasizing the need for tailored strategies to enhance reach and participation. Strong community engagement through ASHAs and village Sarpanches facilitated patient mobilization, improved participation, and strengthened program delivery, particularly in community-based screening.

### Limitation

The study has limitations, including the absence of patient-level data from those who declined enrolment, which prevented the analysis of non-participation factors. Furthermore, the lack of socioeconomic data limited a comprehensive exploration of barriers to reaching the health facility.

## Conclusion

This study highlights the need for scalable, context-specific solutions to reach people with diabetes, particularly in rural areas with limited healthcare access. Effective DRS models combine automated technologies, community-driven strategies, and integrated care to overcome logistical barriers and enable timely diagnosis. Enhancing accessibility, raising awareness, and engaging local stakeholders support sustainable, equitable screening programs that reduce the long-term public health burden of DR.

## Supplementary Information

Supplementary Information.

## Data Availability

The datasets generated and/or analyzed during the current study are not publicly available due to\u0000ethical considerations and confidentiality agreements related to state health systems data\u0000but are available from the corresponding author at a reasonable request.

## References

[1] Raman, R. et al. Prevalence of diabetic retinopathy in India stratified by known and undiagnosed diabetes, urban–rural locations, and socioeconomic indices: Results from the SMART India population-based cross-sectional screening study. *Lancet Glob. Health* **10**(12), e1764–e1773 (2022). 36327997 10.1016/S2214-109X(22)00411-9

[2] Early Photocoagulation for Diabetic Retinopathy. ETDRS report number 9. *Ophthalmology* **98**(5), 766–785. 10.1016/S0161-6420(13)38011-7 (1991). 2062512

[3] Photocoagulation Treatment of Proliferative Diabetic Retinopathy. Clinical Application of Diabetic Retinopathy Study (DRS) Findings, DRS Report Number 8. *Ophthalmology* **88**(7), 583–600. 10.1016/S0161-6420(81)34978-1 (1981). 7196564

[4] Agarwal, M. et al. Diabetic retinopathy screening guidelines for Physicians in India: position statement by the Research Society for the Study of Diabetes in India (RSSDI) and the Vitreoretinal Society of India (VRSI)-2023. *Int. J. Diabetes Dev. Ctries* **44**(1), 32–39 (2024).

[5] Directorate General of Health Services Ministry of Health and Family Welfare Government of India. National program for prevention and control of cancer, diabetes, cardiovascular diseases and stroke (NPCDCS) Directorate General of Health Services Ministry of Health and Family Welfare Government of India Handbook for counselors reducing risk factors for noncommunicable diseases risk factors for NCDs. [cited 2023 Oct 11]. https://main.mohfw.gov.in/sites/default/files/Handbook%20for%20Counselors%20-%20Reducing%20Risk%20Factors%20for%20NCDs_1.pdf.

[6] Raman, R., Ramasamy, K., Rajalakshmi, R., Sivaprasad, S. & Natarajan, S. Diabetic retinopathy screening guidelines in India: All India Ophthalmological Society diabetic retinopathy task force and Vitreoretinal Society of India Consensus Statement. *Indian J. Ophthalmol.* **69**(3), 678–688 (2021). 33269742 10.4103/ijo.IJO_667_20 PMC7942107

[7] Jones, S. & Edwards, R. T. Diabetic retinopathy screening: A systematic review of the economic evidence. *Diabet. Med.* **27**(3), 249–256. 10.1111/j.1464-5491.2009.02870.x (2010). 20536486 10.1111/j.1464-5491.2009.02870.x

[8] Swanson, M. Retinopathy screening in individuals with type 2 diabetes: who, how, how often, and at what cost—An epidemiologic review. *Optometry* **76**(11), 636–646 (2005). 16298316 10.1016/j.optm.2005.08.019

[9] Rushforth, B., McCrorie, C., Glidewell, L., Midgley, E. & Foy, R. Barriers to effective management of type 2 diabetes in primary care: Qualitative systematic review. *Br. J. Gen. Pract.* **66**(643), e114–e127 (2016). 26823263 10.3399/bjgp16X683509 PMC4723210

[10] Hassan, S. et al. A qualitative study exploring the barriers and facilitators of implementing a cardiovascular disease risk reducing intervention for people with severe mental illness into primary care contexts across England: the ‘PRIMROSE’ trial. *BMC Health Serv. Res.* **20**(1), 753. 10.1186/s12913-020-05643-2 (2020). 32799925 10.1186/s12913-020-05643-2 PMC7429749

[11] Ministry of Health and Family Welfare G of I. Operational Guidelines, Eye Care at Health and Wellness Centres (Part of Comprehensive Primary Health Care). [cited 2024 Nov 8]. https://ab-hwc.nhp.gov.in/.

[12] International Agency for the Prevention of Blindness (IAPB). 6. International Agency for the Prevention of Blindness (IAPB). IAPB Vision Atlas 2016. [cited 2023 Nov 9]. http://atlas.iapb.org/vision-trends/diabetic-retinopathy/.

[13] Powell, B. J. et al. Methods to improve the selection and tailoring of implementation strategies. *J. Behav. Health Serv. Res.* **44**(2), 177–194. 10.1007/s11414-015-9475-6 (2017). 26289563 10.1007/s11414-015-9475-6 PMC4761530

[14] Presseau, J. et al. Cluster randomised controlled trial of a theory-based multiple behaviour change intervention aimed at healthcare professionals to improve their management of type 2 diabetes in primary care. *Implement. Sci.* **13**(1), 65. 10.1186/s13012-018-0754-5 (2018). 29720209 10.1186/s13012-018-0754-5 PMC5930437

[15] Nishantha Piyasena, M. M. P. et al. Systematic review on barriers and enablers for access to diabetic retinopathy screening services in different income settings. *PLoS ONE* **14**(4), e0198979 (2019). 31013274 10.1371/journal.pone.0198979 PMC6478270

[16] Rajalakshmi, R., Prathiba, V., Rani, P. K. & Mohan, V. Various models for diabetic retinopathy screening that can be applied to India. *Indian J. Ophthalmol. NLM (Medline)* **69**, 2951–2958 (2021). 10.4103/ijo.IJO_1145_21 PMC8725090 34708729

[17] Glasgow, R. E., Vogt, T. M. & Boles, S. M. Evaluating the public health impact of health promotion interventions: The RE-AIM framework. *Am. J. Public Health* **89**(9), 1322–1327 (1999). 10474547 10.2105/ajph.89.9.1322 PMC1508772

[18] Hoffmann, T. C. et al. Better reporting of interventions: Template for intervention description and replication (TIDieR) checklist and guide. *BMJ* **348**, g1687 (2014). 24609605 10.1136/bmj.g1687

[19] Godwin, M. et al. Pragmatic controlled clinical trials in primary care: the struggle between external and internal validity. *BMC Med. Res. Methodol.* **3**(1), 28 (2003). 14690550 10.1186/1471-2288-3-28 PMC317298

[20] Duggal, M. et al. Protocol paper: Comparing the implementation of different diabetic retinopathy screening models in primary health care settings in Northern India: pragmatic three-arm observational study. *Ser. Endocrinol. Diabetes Metab.* **6**(1), 1–15 (2024).

[21] Government of Punjab I. Basic Statistics of Punjab. [cited 2023 Oct 15]. https://punjab.gov.in/state-profile/.

[22] Ministry of Health and Family Welfare G of I. Rural Health Statistics. [cited 2023 Oct 15]. https://main.mohfw.gov.in/?q=newshighlights-90.

[23] Shi, C. et al. Assessment of image quality on color fundus retinal images using the automatic retinal image analysis. *Sci. Rep.* **12**(1), 10455 (2022). 35729197 10.1038/s41598-022-13919-2 PMC9213403

[24] Shah, P. et al. Validation of Deep Convolutional Neural Network-based algorithm for detection of diabetic retinopathy-Artificial intelligence versus clinician for screening. *Indian J. Ophthalmol.* **68**(2), 398–405 (2020). 31957737 10.4103/ijo.IJO_966_19 PMC7003578

[25] Darwish, D. Y. et al. Diagnostic accuracy and reliability of retinal pathology using the Forus 3nethra fundus camera compared to ultra wide-field imaging. *Eye (Basingstoke)* **33**, 856–857 (2019). 10.1038/s41433-019-0339-9 PMC6707264 30679873

[26] Sosale, B., Sosale, A., Murthy, H., Sengupta, S. & Naveenam, M. Medios-An offline, smartphone-based artificial intelligence algorithm for the diagnosis of diabetic retinopathy. *Indian J. Ophthalmol.* **68**(2), 391–395 (2020). 31957735 10.4103/ijo.IJO_1203_19 PMC7003589

[27] Rajalakshmi, R., Subashini, R., Anjana, R. M. & Mohan, V. Automated diabetic retinopathy detection in smartphone-based fundus photography using artificial intelligence. *Eye (Basingstoke).* **32**(6), 1138–1144 (2018). 10.1038/s41433-018-0064-9 PMC5997766 29520050

[28] Natarajan, S., Jain, A., Krishnan, R., Rogye, A. & Sivaprasad, S. Diagnostic accuracy of community-based diabetic retinopathy screening with an offline artificial intelligence system on a smartphone. *JAMA Ophthalmol.* **137**(10), 1182–1188 (2019). 31393538 10.1001/jamaophthalmol.2019.2923 PMC6692680

[29] The Royal College of Ophthalmologists Diabetic Retinopathy Guidelines. 2012. www.rcophth.ac.uk.

[30] Wong, T. Y. et al. Guidelines on diabetic eye care: The international council of ophthalmology recommendations for screening, follow-up, referral, and treatment based on resource settings. *Ophthalmology* **125**, 1608–1622 (2018). 29776671 10.1016/j.ophtha.2018.04.007

[31] Gadkari, S. S., Maskati, Q. B. & Nayak, B. K. Prevalence of diabetic retinopathy in India: The All India Ophthalmological Society Diabetic Retinopathy Eye Screening Study 2014. *Indian J. Ophthalmol.* **64**(1), 38–44 (2016). 26953022 10.4103/0301-4738.178144 PMC4821119

[32] Kim, E. et al. Impact of individual counseling on the knowledge and attitudes of type 2 diabetics regarding diabetic retinopathy: The Aditya Jyot Diabetic Retinopathy in Urban Mumbai Slums Study—Report 3. *Indian J. Ophthalmol.* **71**(2), 350–356 (2023). 36727318 10.4103/ijo.IJO_1231_22 PMC10228900

[33] StataCorp. 2021. Stata Statistical Software: Release 17. College Station, TX: StataCorp LLC. https://www.stata.com/.

[34] Senjam, S. S. Diabetes and diabetic retinopathy: The growing public health concerns in India. *Lancet Glob. Health* **12**, e727–e728 (2024). 38430917 10.1016/S2214-109X(24)00075-5

[35] Siegler, E. L., Lama, S. D., Knight, M. G., Laureano, E. & Reid, M. C. Community-based supports and services for older adults: A primer for clinicians. *J. Geriatr.* **2015**, 1–6 (2015). 10.1155/2015/678625 PMC4339950 25729774

[36] Chauhan, A. et al. Exploring patient and health care provider perspectives on barriers to diabetic retinopathy screening in public health facilities in North India. *Sci. Rep.* **15**(1), 8251 (2025). 40065058 10.1038/s41598-025-92795-y PMC11894220

[37] Piyasena, M. M. P. N. et al. Systematic review and meta-analysis of diagnostic accuracy of detection of any level of diabetic retinopathy using digital retinal imaging. *Syst. Rev.* **7**(1), 182 (2018). 30404665 10.1186/s13643-018-0846-y PMC6222985

[38] Basu, P. et al. A pilot study to evaluate home-based screening for the common non-communicable diseases by a dedicated cadre of community health workers in a rural setting in India. *BMC Public Health* **19**(1), 14 (2019). 30606132 10.1186/s12889-018-6350-4 PMC6318877

[39] Ministry of Health and Family Welfare G of I. Operational Guidelines for Primary Eye Care at Health and Wellness Centre (A Part of Comprehensive Primary Health Care). [cited 2024 Nov 8]. https://ab-hwc.nhp.gov.in/.

[40] Chauhan, A., Bascaran, C. & Duggal, M. Effects of the quality of power supply on diabetic retinopathy screenings: An experience from India. *J. Vis. Impair Blind.* **116**(6), 850–852. 10.1177/0145482X221144447 (2022).

[41] Mestre, F., Mendes De Melo, T., De Freitas Alvarenga, K., Quinhoneiro Blasca, W. & Frederico De Lima Taga, M. Community health agents training on hearing health: Effectiveness of videoconference (2010). 10.1590/s0104-56872010000200012 20640378

[42] Gulshan, V. et al. Development and validation of a deep learning algorithm for detection of diabetic retinopathy in retinal fundus photographs. *JAMA* **316**(22), 2402–2410. 10.1001/jama.2016.17216 (2016). 27898976 10.1001/jama.2016.17216

[43] Abràmoff, M. D., Lavin, P. T., Birch, M., Shah, N. & Folk, J. C. Pivotal trial of an autonomous AI-based diagnostic system for detection of diabetic retinopathy in primary care offices. *NPJ Digit. Med.* **1**(1), 39. 10.1038/s41746-018-0040-6 (2018). 31304320 10.1038/s41746-018-0040-6 PMC6550188

[44] Sengupta, S., Sindal, M. D., Baskaran, P., Pan, U. & Venkatesh, R. Sensitivity and specificity of smartphone-based retinal imaging for diabetic retinopathy: A comparative study. *Ophthalmol. Retina* **3**(2), 146–153 (2019). 31014763 10.1016/j.oret.2018.09.016

[45] University of North Carolina at Chapel Hill. Hospital Ergonomics. [cited 2025 Mar 30]. https://ehs.unc.edu/topics/ergonomics/hospital-ergonomics/.

[46] Prathiba, V. et al. Accuracy of the smartphone-based nonmydriatic retinal camera in the detection of sight-threatening diabetic retinopathy. *Indian J. Ophthalmol.* **68**(13), S42–S46 (2020). 31937728 10.4103/ijo.IJO_1937_19 PMC7001191

[47] Gadkari, S. S. Diabetic retinopathy screening: Telemedicine, the way to go!. *Indian J. Ophthalmol.* **66**, 187–188 (2018). 29380754 10.4103/ijo.IJO_1155_17 PMC5819091

[48] Lau, R. et al. Achieving change in primary care-causes of the evidence to practice gap: Systematic reviews of reviews. *Implement Sci.* **11**, 40 (2016). 27001107 10.1186/s13012-016-0396-4 PMC4802575

[49] Ministry of Health & Family Welfare National Programme For Prevention And Control Of Non-Communicable Diseases Operational Guidelines National Programme for Prevention and Control of Non-Communicable Diseases (2023).

[50] Directorate General of Health Services Ministry of Health and Family Welfare Government of India. National Programme for Prevention and Control of Cancer, Diabetes, Cardiovascular Diseases and Stroke (NPCDCS), Directorate General of Health Services Ministry of Health and Family Welfare Government of India Handbook for counselors reducing risk factors for noncommunicable diseases risk factors for NCDs.

