# Supplementary material for: Reach and implementation of human and AI-assisted diabetic retinopathy screening models in primary healthcare settings in India
Source: Sci Rep. 2025 Nov 21;15:41355. doi: 10.1038/s41598-025-25402-9 (PMC12638931; doi:10.1038/s41598-025-25402-9)
Supplement: Supplementary file 1 — Supplementary Information. [file 41598_2025_25402_MOESM1_ESM.docx]

**Reach and Implementation of Human and AI Assisted Diabetic Retinopathy Screening Models in Primary Healthcare Settings in India**

Supplementary Table 1: The TIDieR (Template for Intervention Description and Replication) Checklist

| **Item No** | **Item** | **Description** | **Section of the manuscript**  **Page no** |
| --- | --- | --- | --- |
| **1** | **Brief name**  Provide the name or a phrase that describes the intervention. | 1a: Diabetic retinopathy screening (DRS)  1b: Three-arm pragmatic trial: arm I - non-ophthalmologist-based DRS at HWC, Arm II - AI-based DRS in the community settings (home), Arm III - standard care in the community settings. | Study design and settings and intervention description, page 6 |
| **2** | **Why**  Describe any rationale, theory, or goal of the elements essential to the intervention | 2a: Diabetic retinopathy (DR) often progresses silently to vision-threatening stages, risking severe vision impairment in 26% within two years without treatment. Early screening for timely detection and management of DR is critical, given diabetes's high prevalence.(1,2)  2b: The study applied the RE-AIM implementation research framework to compare the implementation process of the DRS intervention. This approach enhances the generalizability and translation of evidence-based interventions into sustained services. (3,4)  2c: The pragmatic three-arm observational study allowed for evaluation reach, implementation, and adoption in real-world public health settings, which increases the external validity and is critical for generalisability or research findings. (5–7) | Study design and settings and intervention description, page 6  See Protocol paper for more details: Comparing the Implementation of Different Diabetic Retinopathy Screening Models in Primary Health Care Settings in Northern India: Pragmatic Three-arm Observational Study (8) |
| **3** | **What**  Materials: Describe any physical or informational materials used in the intervention, including those provided to participants or used in intervention delivery or in training of intervention providers. Provide information on where the materials can be accessed (e.g. online appendix, URL). | The activities under the Reach and implementation to conduct the DRS is explained:  **Reach:** It explains how to identify the population to be reached and benefited by the interventions (PwDM), including line listing, mapping, and stakeholder sensitization.  **Implementation:** refers to the extent to which intervention agents adhere to an intervention's different components, including the intervention's delivery and timing. This includes preparatory steps, conducting DRS in three arms, grading, referral, counseling, and linking to referral centers. | Study design and settings and intervention description, page 6  procedures from reach to linking to referral centres page 6-12  Procedures explaining the process of Reach (R) and implementation (I) of DRS in the given study are available in the protocol paper (8) |
| **4** | Procedures: Describe each of the procedures, activities, and/or processes used in the intervention, including any enabling or support activities. | - **Reach:** line listing, mapping, stakeholder sensitization.   Line listing: includes enumerating all the households that detail the information of the head of the household and the medical history (history of diabetes, hypertension, ocular history) of the family members  Mapping: involves creating a map of the area (village) to facilitate the identification of eligible households during further visits.  Stakeholder sensitization: This involves sensitizing stakeholders about the activities to be conducted before, during, and after the study.   - **Implementation:** preparatory steps, conducting DRS in three arms, grading, referral, counselling and linking to referral centres.   Preparatory steps: Training of non-ophthalmologists to capture fundus imaging in study arms  DRS and grading: non-mydriatic fundus imaging and grading by a human grader and AI  Referral and counselling and linking to referral centres: referral recommendations, (9), counselling on complications of DM, including its signs and symptoms, and the importance of early detection of DR(10) | Study design and settings and intervention description, page 6  procedures from reach to linking to referral centres page 6-12  Procedures explaining the process of Reach (R) and implementation (I) of DRS in the given study are available in the protocol paper (8) |
| **5** | **Who provided**  For each category of intervention provider (e.g., psychologist, nursing assistant), describe their expertise, background, and any specific training given. | Three non-ophthalmologists with a bachelor's degree in optometry acquired the fundus images after a 15-day training program at the PGIMER Advanced Eye Centre (AEC). This training equipped them with the necessary skills for image acquisition. Sensitization meetings at the PHC and village levels introduced the study and sought stakeholder support.  The non-ophthalmologist captured fundus images in arms I and II. | Preparatory steps, page 9 |
| **6** | **How**  Describe the modes of delivery (e.g. face-to-face or by some other mechanism, such as internet or telephone) of the intervention and whether it was provided individually or in a group. | **Fundus imaging modalities:** Two fundus cameras were used for the DRS in two study arms  Fundus benchtop camera for Arm I: The benchtop camera is supported by a laptop running MS Windows 10 and above, with a 64-bit operating system and an i3 10th-generation processing system. Forus 3nethra classic (benchtop) camera (11,12) was used to capture images at the HWC.  Smartphone-based fundus camera for Arm II: This camera can be used as a handheld device or with its mounted stand. It is lightweight, portable, and has a power backup for screening in community settings. Remidio NM FOP 10 (smartphone-based handheld) integrated cameras with the offline Medios AI algorithm (13,14) was used in the community settings.  **Diabetic retinopathy grading**  Human graders in arm I and Offline AI grading (Medios) in arm II (13,15) | Diabetic retinopathy grading page 10  Imaging modalities details are available in the protocol paper (8) |
| **7** | **Where**  Describe the type(s) of location(s) where the intervention occurred, including any necessary infrastructure or relevant features. | This three-arm observation study was conducted in the primary healthcare settings as described below:  Arm I: HWC Khijrabad, Block Boothgarh, District Mohali, Punjab, India  Arm II: Community settings (home), Block Boothgarh, District Mohali, Punjab, India  Arm III: Community settings (home), Block Boothgarh, District Mohali, Punjab, India  Study design and settings and intervention description, page 6  The details are available in the protocol paper (8) | Study design and settings and intervention description, page 6  The details are available in the protocol paper (8) |
| **8** | **When and how much**  Describe the number of times the intervention was delivered and over what period of time including the number of sessions, their schedule, and their duration, intensity or dose | The DRS was performed as a one-time intervention, with referral recommendations provided accordingly. | Study design and settings and intervention description, page 6 |
| **9** | **Tailoring**  If the intervention was planned to be personalised, titrated or adapted, then describe what, why, when, and how. | The implementation domain of the RE-AIM framework highlights the challenges encountered and adaptations made during the delivery of DRS in HWCs and community settings (home). | The what, why, when, and how of the Tailoring of the intervention is described in results section, Challenges and adaptations during the implementation of DRS models, Page 15-16 |
| **10** | **Modifications**  If the intervention was modified during the course of the study, describe the changes (what, why, when, and how). | The implementation domain of the RE-AIM framework highlights the challenges encountered and adaptations made during the delivery of DRS in HWCs and community settings (home). | The what, why, when, and how of the Tailoring of the intervention is described in results section, Challenges and adaptations during the implementation of DRS models, Page 15-16 |
| **11** | **How well**  Planned: If intervention adherence or fidelity was assessed, describe how and by whom, and if any strategies were used to maintain or improve fidelity, describe them. | This manuscript focuses on evaluating the Reach and Implementation components of the RE-AIM framework. We have presented the referral recommendations and linking to referral centres. The Adoption aspect, which examines adherence to referral instructions, will be addressed in a separate manuscript  Adoption in the present study is defined as 1) Total referral recommendations provided through each DRS intervention and rate of adherence and 2) stakeholders perceptions: PwDM, research staff, healthcare providers (HCP), and program officers regarding the usability and acceptability of the DRS interventions. | Study design and settings and intervention description, page 6 |
| **12** | Actual: If intervention adherence or fidelity was assessed, describe the extent to which the intervention was delivered as planned. | The adoption domain of the RE-AIM framework will be discussed in another manuscript. | The description on the coverage of the RE-AIM framework are available in the protocol paper (8) |

AI: Artificial intelligence, DRS: Diabetic retinopathy screening, HWC: Health and wellness centre, PGIMER: Post Graduate Institute of Medical Education and Research

Supplementary Table 2: Summary characteristics of Punjab and district Mohali

| **Characteristics** | **Punjab** | **Mohali** |
| --- | --- | --- |
| **Basic statistics of Punjab** | | |
| **Population (n)** | 27704000 | 990000 |
| Rural n (%) | 17317770 (62.51%) | 442112 (44.8%) |
| Urban n (%) | 10386230 (37.49%) | 544035 (55.2%) |
| **Villages (n)** | 12081 | 423 |
| **Health facilities** | | |
| HWC | 2951 | 76 |
| PHC | 522 | 19 |
| CHC | 160 | 5 |
| DH/SDH | 42 | 1 |
| **Ophthalmologist** | - | 1 |
| **Optometrist** | - | - |

The data in the table is obtained from the references (16–18)

CHC- Community Health Centre, DH- District Hospital, PHC- Primary Health Centre, SDH- Sub-District Centre

Supplementary Table 3: The existing process of DM management and DRS in public health facilities

| **Activity** | **Primary health facilities** | **Secondary health facilities** | **Tertiary health facilities** |
| --- | --- | --- | --- |
| Diabetes diagnosis | Yes | Yes | Yes |
| Treatment of DM | Yes | Yes | Yes |
| Education on DR | Yes | Yes | Yes |
| DRS and grading | No | No | Yes |
| Laser treatment | No | No | Yes |
| Anti-VEGF injection | No | No | Yes |
| PPV | No | No | Yes |

Primary- HWC, PHC; Secondary - CHC, DH; Tertiary- tertiary hospital, PPV – Pars Plan Vitrectomy, VEGF - Vascular Endothelial Growth Factors

Red - Non-availability of services, Green - availability of services

Supplementary Figure 1: Screening pathway in study arms


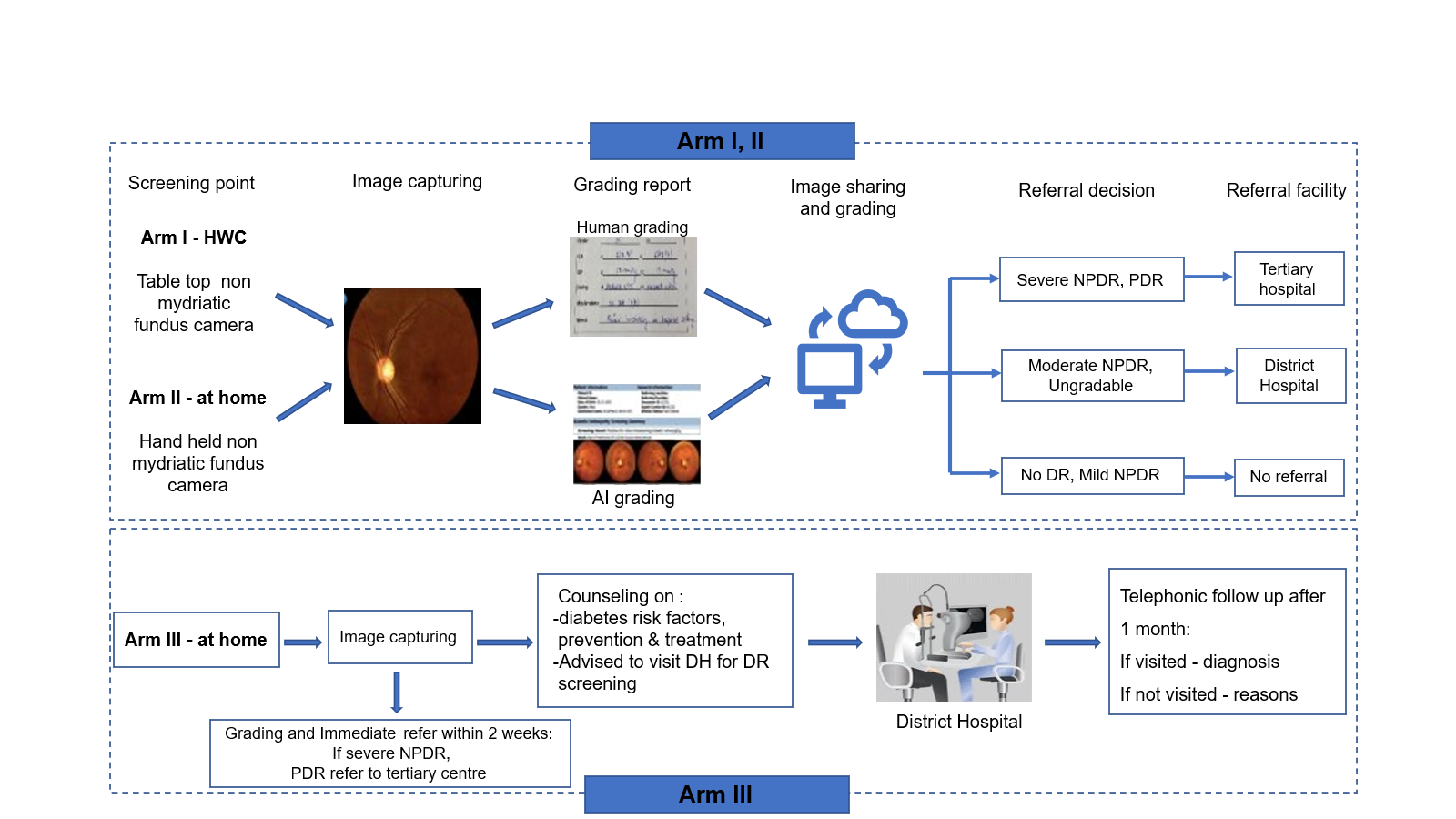


DR: Diabetic retinopathy, HWC: Health and Wellness Centre, NPDR: Non-proliferative diabetic retinopathy, PDR: Proliferative diabetic retinopathy

Source: Duggal Mona, Chauhan A, Gupta et al. Protocol Paper: Comparing the implementation of different diabetic retinopathy screening models in primary health care settings in Northern India: a pragmatic three-arm observational study. Series of Endocrinology, Diabetes and Metabolism [Internet]. 2024 Apr 30 [cited 2024 Oct 28];6(1):1–15. Available from: https://seriesscience.com/diabetic-retinopathy-screening/

Supplementary Figure 2: Village map showing line-listed households


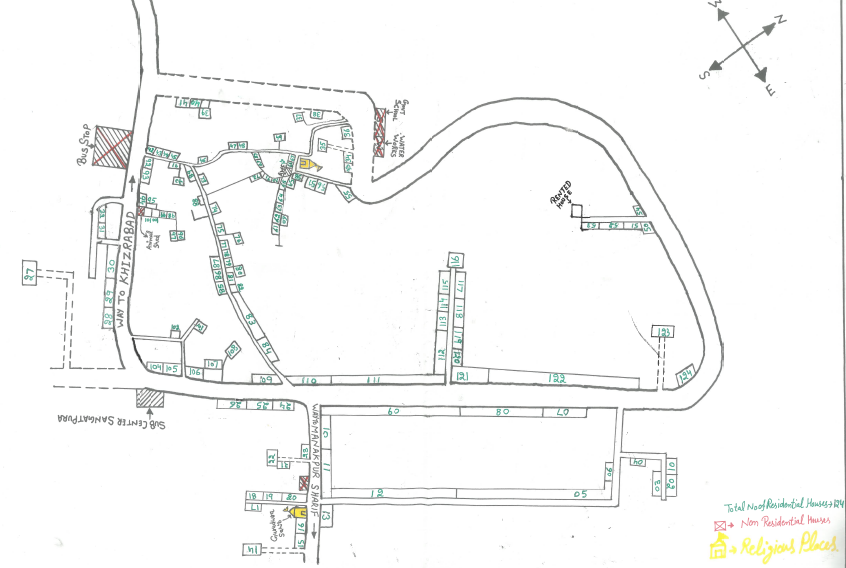


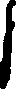

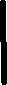

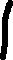

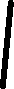


Supplementary Table 4: Key informants roles and responsibilities

| **Group** | **Key informant** | **Place of posting** |
| --- | --- | --- |
| Health care providers (HCP) | **Punjab Health Department** | |
|  | Medical officer | Primary Health Centre (PHC) |
|  | ASHA | Community (villages) |
|  | CHO | Health and Wellness Centre (HWC) |

ASHA: Accredited Social Health Activist, PGIMER: Post Graduate Institute of Medical Education and Research, HCP: Health Care Providers, Health and Wellness Centre (HWC) PHC: Primary Health Centre

Supplementary Figure 3 (a-b): Original interior pictures of the screening room at the HWC Khijrabad


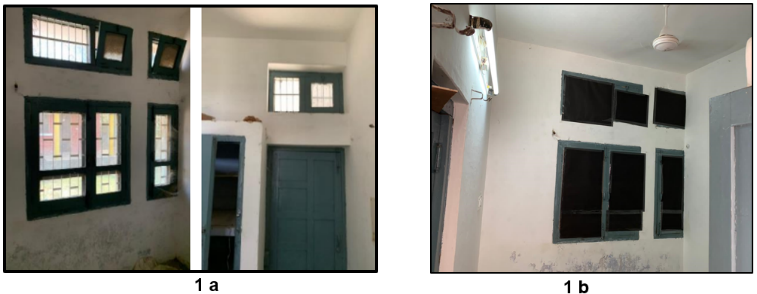


Supplementary Figure 3 (c-d): Established dark room at the HWC Khijrabad


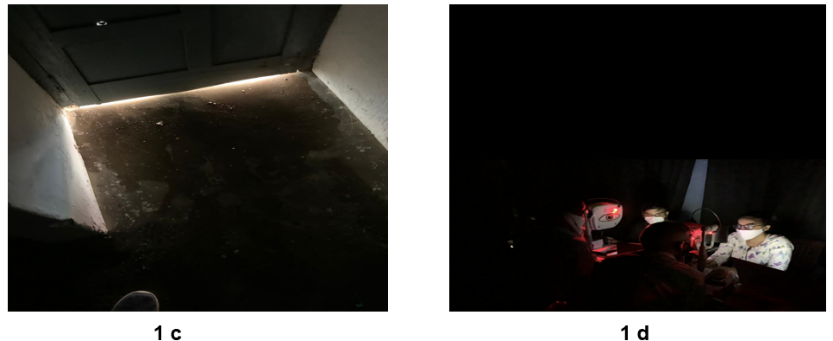


Supplementary Table 5: Sociodemographic Characteristics of the Study Participants (Arm-wise and Overall)

| **Characteristics** | **Overall**  **(N=200)** | **Arm I**  **(HWC, N=200)** | **Arm II**  **(Community, N=200)** | **Arm III**  **(Community, N=200)** |
| --- | --- | --- | --- | --- |
| **Age (years),**  **Mean ± SD** | 58.22(11.52) | 57.9 (11.41) | 58.22 (11.45) | 58.55 (11.74) |
| **Age (years),**  **Median (IQR)** | 59 (50-65) | 56.5 (50-65) | 60 (50-67) | 60 (50-65) |
| Age categories (years), n(%) |  |  |  |  |
| ≤ 40 | 46 (7.65) | 10 (5) | 19 (9.5) | 17 (8.46) |
| 41 - 50 | 119 (19.80) | 53 (26.5) | 32 (16) | 34 (16.92) |
| 51 - 60 | 181 (30.12) | 56 (28) | 61 (30.5) | 64 (31.84) |
| 61 - 70 | 177 (29.45) | 56 (28) | 64 (32) | 57 (28.36) |
| >70 | 78 (12.98) | 25 (12.5) | 24 (12) | 29 (14.43) |
| Gender, n (%) |  |  |  |  |
| Male | 245 (40.77) | 84 (42) | 73 (36.5) | 88 (43.78) |
| Female | 355 (59.23) | 116 (58) | 127 (63.5) | 113 (56.22) |
| Diabetes duration, (years) n (%) |  |  |  |  |
| ≤5 | 286 (48.31) | 96 (50) | 92 (46.23) | 98 (48.76) |
| 5.1–10 | 170 (28.72) | 53 (27.6) | 62 (31.16) | 55 (27.36) |
| 10.1–15 | 89 (15.03) | 29 (15.1) | 28 (14.07) | 32 (15.92) |
| ≥15.1 | 47 (7.94) | 14 (7.29) | 17 (8.54) | 16 (7.96) |

*HWC-Health and Wellness Centre, IQR-Interquartile range, SD-Standard deviation

Supplementary Table 6: Implementation activities in each study arm at different sites

| **Activities** | **Activity description** | **Arm I**  **(HWC)** | **Arm II**  **(Community)** | **Arm III**  **(Community)** |
| --- | --- | --- | --- | --- |
| **LL & mapping** | Total HH (n) | 2042 | 1124 | 1466 |
|  | Total pop (n) | 12687 | 6819 | 6514 |
|  | Per day HH coverage (n) | 52 | 51 | 56 |
|  | Total HH coverage (days) | 39 | 22 | 26 |
|  | **Per day HH activity** |  |  |  |
|  | -Introduction & study  brief (min) | 10 | 10 | 10 |
|  | -HH numbering (min) | 4 | 4 | 4 |
|  | -Data capturing (min) | 12 | 12 | 12 |
|  | Form entry & maps (days) | 20 | 11 | 14 |
|  | Human resource (n) | 3 | 3 | 3 |
| **Enrolment:**  **Invitation & appointment** | **Day 2: Consent and appointment (min)** | 5 | 5 | 5 |
|  | Human resource (n) | 2 | 2 | 2 |
| **Diabetic retinopathy screening** | **Day 3: Screening activities** |  |  |  |
|  | CEE (min) | 5-6 | 10 | 10 |
|  | NMFI (min) | 6-7 | 10-12 |  |
|  | Data capturing (min) | 7 | 11-12 | 11-12 |
|  | Counselling (min) | 4 | 4 | 4 |
|  | Screening/day | 10-12 | 7-8 | 7-8 |
|  | Total activity time for each participant (min) | 28 | 48 | 48 |
|  | Total screening days,  200 participants (days) | 21 | 32 | 34 |
|  | Human resource (n) | 3 | 4 | 4 |
| **Reference grading** | Per day participant image grading | 12 | 12 | 12 |
|  | Total set grading (days) | 17 | 17 | 17 |
| **Grading result distribution** | ASHA network (days) | 7 | 7 | 0 |
| **Telephonic follow-up** | Number of days (n) | 8 | 8 | 17 |
|  | Human resource (n) | 1 | 1 | 1 |

Abbreviations: ASHA: Accredited social health activist, CEE: Comprehensive eye examination, HH: Household, HWC: Health and wellness centre, NMFI: Non-mydriatic fundus imaging

Supplementary Table 7: Reasons for refusals in study arms underreach

| **Arm I** | **Arm II** | **Arm III** |
| --- | --- | --- |
| No traceable HH-7  Non-diabetics - 3  Incomplete data - 2  Not interested - 15  HH locked - 18  Other medical conditions - 12  Unable to cooperate - 10  Refused to image - 6  Out of the station - 4  ECHS card - 1  Injury/conjunctivitis/redness - 5  Travel distance to PHC - 38  Felt eyes are fine - 2  death - 1  work commitment - 5  cataract operated - 1 Incomplete image folder - 5 | No traceable HH - 2  Non-diabetics - 1  Incomplete data - 1  Not interested - 5  Work commitment - 2  HH locked - 5  Unable to cooperate - 8  Refused imaging - 5 Redness/watery eyes - 2  Incomplete image folder - 2 | No traceable HH - 2  Non-diabetics - 1  Incomplete data - 1  Not interested - 4  Work commitment - 3  HH locked - 5  Unable to cooperate - 4  Other diseases -1  Refused imaging - 3 Redness/watery eyes - 1  Incomplete image folder - 4 |

Abbreviations: HH: Household

Supplementary Table 8: Representative quotes to describe chgallanges and adaptation during DRS implementation in study arms

| **Theme** | **Sample of illustrative quotes** |
| --- | --- |
| **Barriers to screening accessibility and community outreach** | *"Due to my old age, I can't visit the facility on my own and have to depend on my family members to take me for screening,"* *said a patient (P1).* |
|  | *"The screening facility is 5 kilometers from my home, and with no public transport available, I have to rely entirely on my family, who are often away at work,"* *shared another patient (P3).* |
|  | *“Based on my experience, most patients do not attend screenings either because they do not perceive it as an urgent need or due to the distance from the facility.”* *ASHA-1* |
|  | *"Community engagement is critical for implementing healthcare programs. The sensitization meetings helped us engage and encourage people to attend the screening," MO1* |
|  | *"The success of public health programs lies in an integrated approach where we move together, involving the patient, the community, and the health system." CHO3* |
| **Ergonomic barriers and workflow optimization** | *"Doctors and operators often discuss ergonomics, but we must also focus on patient ergonomics. If the patient isn't seated properly, capturing high-quality non-mydriatic images becomes difficult." Optom1* |
|  | *"We relocated adjustable chairs and a height-adjustable table for the camera from the Advanced Eye Centre to ensure optimal screening conditions and facilitate a smooth screening process." Optom2* |
|  | *"We promptly arranged a short training session for the optometrist, conducted by a certified retina technician at the Advanced Eye Centre, to ensure proper techniques for patient handling, transferring, and positioning, reducing the risk of injuries from awkward postures and ergonomic stressors." PI* |
| **Addressing infrastructure & equipment limitations** | *"We must adapt to the program's needs and strengthen the health system’s infrastructure, just as we did by installing a generator and buying adapters immediately to keep the screening ongoing."* *PI* |
|  | *"To keep the screening program running smoothly, we brought a camera engineer on board for the screening period, ensuring that hardware issues were resolved without delays," explained a program implementer.” PI* |
| **Optimizing image quality through darkroom adaptations** | *"Creating a dedicated darkroom for screening was a crucial intervention, enabling us to capture high-quality non-mydriatic images." Optom and Opt* |
|  | *"This approach holds significant potential for public health screenings, particularly in settings where pupil dilation may not always be practical." Optom and Opt* |

**References**

1. Early Photocoagulation for Diabetic Retinopathy: ETDRS Report Number 9. Ophthalmology [Internet]. 1991 May 1;98(5):766–85. Available from: https://doi.org/10.1016/S0161-6420(13)38011-7

2. Photocoagulation Treatment of Proliferative Diabetic Retinopathy: Clinical Application of Diabetic Retinopathy Study (DRS) Findings, DRS Report Number 8. Ophthalmology [Internet]. 1981 Jul 1;88(7):583–600. Available from: https://doi.org/10.1016/S0161-6420(81)34978-1

3. Glasgow RE, Harden SM, Gaglio B, Rabin B, Smith ML, Porter GC, et al. RE-AIM planning and evaluation framework: Adapting to new science and practice with a 20-year review. Vol. 7, Frontiers in Public Health. Frontiers Media S.A.; 2019.

4. Welch A, Healy G, Straker L, Comans T, O’Leary S, Melloh M, et al. Process evaluation of a workplace-based health promotion and exercise cluster-randomised trial to increase productivity and reduce neck pain in office workers: A RE-AIM approach. BMC Public Health. 2020 Feb 4;20(1).

5. Roland M, Torgerson DJ. Understanding controlled trials What are pragmatic trials?

6. Godwin M, Ruhland L, Casson I, Macdonald S, Delva D, Birtwhistle R, et al. Pragmatic controlled clinical trials in primary care: the struggle between external and internal validity [Internet]. 2003. Available from: http://www.biomedcentral.com/1471-2288/3/28

7. Patsopoulos NA. A pragmatic view on pragmatic trials [Internet]. Vol. 13, Dialogues Clin Neurosci. 2011. Available from: www.dialogues-cns.orgPAGES_12_AG_1003_BA.qxd:DCNS#4931/05/110:16Page217

8. Duggal Mona, Chauhan A, Gupta et al. Protocol Paper: Comparing the implementation of different diabetic retinopathy screening models in primary health care settings in Northern India: pragmatic three-arm observational study. Series of Endocrinology, Diabetes and Metabolism [Internet]. 2024 Apr 30 [cited 2024 Oct 28];6(1):1–15. Available from: https://seriesscience.com/diabetic-retinopathy-screening/

9. Wilkinson CP, Ferris FL, Klein RE, Lee PP, Agardh CD, Davis M, et al. Proposed international clinical diabetic retinopathy and diabetic macular edema disease severity scales. Ophthalmology [Internet]. 2003;110(9):1677–82. Available from: https://www.sciencedirect.com/science/article/pii/S0161642003004755

10. Kim E, Rana V, Araia E, Jain A, Krishnan R, Natarajan S. Impact of individual counseling on the knowledge and attitudes of type 2 diabetics regarding diabetic retinopathy: The Aditya Jyot Diabetic Retinopathy in Urban Mumbai Slums Study - Report 3. Indian J Ophthalmol. 2023 Feb 1;71(2):350–6.

11. Shah P, Mishra D, Shanmugam M, Doshi B, Jayaraj H, Ramanjulu R. Validation of Deep Convolutional Neural Network-based algorithm for detection of diabetic retinopathy-Artificial intelligence versus clinician for screening. Indian J Ophthalmol. 2020 Feb 1;68(2):398–405.

12. Darwish DY, Patel SN, Gao Y, Bhat P, Chau FY, Lim JI, et al. Diagnostic accuracy and reliability of retinal pathology using the Forus 3nethra fundus camera compared to ultra wide-field imaging. Vol. 33, Eye (Basingstoke). Nature Publishing Group; 2019. p. 856–7.

13. Sosale B, Sosale A, Murthy H, Sengupta S, Naveenam M. Medios-An offline, smartphone-based artificial intelligence algorithm for the diagnosis of diabetic retinopathy. Indian J Ophthalmol. 2020 Feb 1;68(2):391–5.

14. Rajalakshmi R, Subashini R, Anjana RM, Mohan V. Automated diabetic retinopathy detection in smartphone-based fundus photography using artificial intelligence. Eye (Basingstoke). 2018 Jun 1;32(6):1138–44.

15. Sundaram Natarajan 1 AJ 1, RK 1, AR 1, SS 2. Diagnostic Accuracy of Community-Based Diabetic Retinopathy Screening With an Offline Artificial Intelligence System on a Smartphone. JAMA Ophthalmol [Internet]. [cited 2023 Oct 17]; Available from: https://www.ncbi.nlm.nih.gov/pmc/articles/PMC6692680/

16. Government of Punjab I. Basic Statistics of Punjab. [cited 2023 Oct 15]; Available from: https://punjab.gov.in/state-profile/

17. Office of Registrar General & Census Commissioner I, Ministry of Home Affairs G of I. Primary Census Abstract. [cited 2023 Oct 15]; Available from: https://censusindia.gov.in/census.website/data/census-tables#collapseExample_0

18. Ministry of Health and Family Welfare G of I. Rural Health Statistics. [cited 2023 Oct 15]; Available from: https://main.mohfw.gov.in/?q=newshighlights-90
